# The Autoimmune Model of Schizophrenia

**DOI:** 10.5402/2012/758072

**Published:** 2012-04-30

**Authors:** D. D. Adams, J. G. Knight, A. Ebringer

**Affiliations:** ^1^Faculty of Medicine, University of Otago, Box 913, Dunedin 9050, New Zealand; ^2^Division of Commerce, University of Otago, Dunedin 9054, New Zealand; ^3^Kimg's College London, University of London, London SE1 8UB, UK

## Abstract

Schizophrenia is of mysterious causation. It is not infectious, not congenital, but shows familial aggregation, the Mendelian genetics indicating involvement of multiple codominant genes with incomplete penetrance. This is the pattern for autoimmune diseases, such as Graves' disease of the thyroid, where forbidden clones of B lymphocytes develop, and cause thyrotoxicosis by secreting autoantibodies that react with the thyroid gland's receptor for thyroid-stimulating hormone from the pituitary gland. In 1982, Knight postulated that autoantibodies affecting the function of neurons in the limbic region of the brain are a possible cause of schizophrenia. Today, this is even more probable, with genes predisposing to schizophrenia having being found to be immune response genes, one in the MHC and two for antibody light chain V genes. Immune response genes govern the immune repertoire, dictating the genetic risk of autoimmune diseases. The simplest test for an autoimmune basis of schizophrenia would be trial of immunosuppression with prednisone in acute cases. The urgent research need is to find the microbial trigger, as done by Ebringer for rheumatoid arthritis and for ankylosing spondylitis. This could lead to prophylaxis of schizophrenia by vaccination against the triggering microbe.

## 1. Introduction

Louis Pasteur's epochal *Germ Theory of Disease *[1] triggered the fastest advance of medicine ever. The great host of infectious diseases, with their aetiology at last discovered, was soon substantially conquered. Vaccinations provided prophylaxis and antibiotics therapy. Additionally, freed of inevitable wound infection, modern surgery was made possible, led by Lister [2]. In stark contrast to the infectious diseases the autoimmune diseases, have been a cinderella of Medicine. Coombs and Gell's classification of harmful immune responses [3] was preautoimmunity, antediluvian, based on the misapprehension that Paul Ehrlich's “horror autotoxicus” dictum was based on proof that autoimmunity never occurs. In reality, Ehrlich [4] wrote that “possible failure of the internal regulation” of immune processes might be “the explanation of many disease phenomena.” The independent demonstration of autoimmunity in four different laboratories [5–9] made Coombs and Gell's classification obviously wrong, yet it lingered on for decades [10] as an inhibition to clarity of thought.

Table 1 shows a valid classification [11] based on awareness of autoimmunity and awareness that allergy is a defect of immune defence against worms and other parasites, being caused by the occurrence of B lymphocyte clones that secrete IgE antibodies with specificity for nonparasitic foreign antigens.

## 2. Pathogenesis of Autoimmune Disease

### 2.1. The Forbidden Clone Theory

Autoimmune diseases are caused by defects of immune specificity, which is determined by the amino acid sequence of the variable regions of the receptors for antigen on B and T lymphocytes, not by cytokines nor dendritic cells. Niels Jerne won the Nobel Prize for his epochal realization that antibodies are not formed on a template of antigen but are preformed, in myriad diversity, awaiting contact with an antigen that fits, the *Selection Theory *[12]. Macfarlane Burnet realised that it is not antibodies that are selected but the cells that make them and that these cells are lymphocytes, whose unit of specificity is the lymphocyte clone, a subset of lymphocytes with identical receptors for antigen, hence, Burnet's *Clonal Selection Theory *[13] of acquired immunity, today in general acceptance. However, widely neglected is Burnet's equally important *Forbidden Clone Theory *[14], postulating that autoimmune disease is caused by somatic mutation in lymphocytes multiplying in response to a microbial antigenic stimulus.

### 2.2. Thyroid-Stimulating Autoantibodies

In 1838, Robert Graves, in Dublin, identified the syndrome of tachycardia, goitre, weight loss and exophthalmos, which bears his name as *Graves' disease* [15]. 

In 1933, Charles Harington isolated *thyroxine* [16], the first hormone to be chemically isolated, and found the molecule to contain iodine atoms, explaining the cause of *endemic goitre,* which is due to deficiency of dietary iodine.

In 1956, Adams and Purves [17, 18] in Dunedin, using radioactive iodine for a bioassay of thyroid-stimulating hormone in baby guinea pigs, discovered a *long-acting thyroid stimulator (LATS)*. This proved to be an autoantibody that causes thyrotoxicosis [19] by accidentally reacting with the thyroid gland receptor for thyroid-stimulating hormone from the pituitary gland, the conductor of the endocrine orchestra.

### 2.3. Confirmation of the Forbidden Clone Theory in Graves' Disease

Affinity chromatography of thyroid-stimulating autoantibodies from individual patients shows that they contain only one of two possible antibody light chain types, K or *λ*, but never both [20]. Therefore, these autoantibodies originate from *single B lymphocytes* in which a V gene has mutated. Furthermore, the variation from patient to patient of reactivity with the slightly differing TSH receptors of various nonhuman animals shows *the random element* in the somatic mutations forming the thyroidstimulating autoantibodies [19].

### 2.4. Suppressor T Cells and Autoimmunity

In 1971 Allison et al. [21] postulated that lack of suppressor T cells causes the autoimmune diseases.

In 1975 Nachigal et al. [22] proposed that on meeting antigen, *mature* T cells become helpers and *immature* T cells become Suppressors.

This limits specific immune responses, preventing both leukemia and autoimmunity.

In 1978 Knight and Adams [23] disproved claims that lack of suppressor T cells causes autoimmune disease in the New Zealand mice.

In 1999 Shevack [24], in Paul's *Fundamental Immunology, *reported that *the autoimmune diseases are not caused by lack of suppressor T cells*.

In 2010 Adams et al. [25] recommended research switch to selective destruction of forbidden clones, with methods available.

### 2.5. The Cause of Diabetic Retinopathy

The retinal vasculature has pericyte cells, supporting the endothelial cells. Klintworth [26] discovered that in diabetic retinopathy there is a loss of pericyte cells.

Adams [27] has postulated that the pericyte loss is the cause of the vascular collapse of diabetic retinopathy. Furthermore, Adams proposes that the pericytes are destroyed by forbidden clones of cytotoxic T cells, antigenically related to those that cause type l diabetes by destroying the pancreatic islet *β* cells that make insulin. This seems to be an example of cross-tissue T cell autoimmunity, analogous to the cross-tissue B cell autoimmunity of Graves' disease, where thyroid cells and orbital fat cells can both be targets, causing thyrotoxicosis and exophthalmos. Therapeutically, this new understanding suggests that ineffective “tight glycaemic control” should be replaced by immunosuppression with low-dose prednisone for treatment of diabetic retinopathy.

## 3. Genetics of Autoimmune Disease

### 3.1. The Major Histocompatibility Complex (MHC)

 Why do we have histocompatibility antigens? The main reason is for defence against the explosive speed of replication of infecting viruses [28]. In influenza virus infection [29], for example, each virus-infected cell bursts open after 18 hours to release about a thousand virions, each of which infects a new cell, which in turn produces another thousand virions and so on, with death of the patient in 3 or 4 days. Zinkernagel and Doherty [30] discovered how this is prevented. They found that peptides, from a virus infecting a cell, are extruded to the cell surface on a histocompatibility antigen molecule. There, the viral peptide is available for reaction with the antigen receptor of a cytotoxic T-cell clone, if one with complementary antigen specificity exists.

Why is the involvement of the histocompatibility molecule necessary? This was first explained in The Lancet in 1987 [29], which shows that the MHC presentation is necessitated by the explosive speed of virus replication. This huge number of virions would swamp the cytotoxic T cells, muffling their antigen receptors and so preventing them from destroying the virus factories that the infected cells become. It follows that to prevent virus infections from being universally lethal, the defending cytotoxic T-cell clones must be reactive with the *conjoint MHC-virus antigen* on the surface of the infected cells, rather than reactive with the free virions.

### 3.2. The H Gene Theory

The Forbidden Clone Theory, postulating somatic mutations in lymphocytes as the cause of the autoimmune diseases, does not explain the familial aggregation, which indicates involvement of germline genes.

When the immunoglobulin V (variable region) genes, coding for the antigen specificity of lymphocyte clones were discovered, they seemed likely to be germline genes that influence the risks of autoimmune diseases. This remains highly probable. However, the discovery that the autoimmune disease lupus nephritis occurs in F l hybrids of two inbred strains of mice, but not in either parental strain, afforded an opportunity to analyse the parental contributions to this autoimmune disease. In Dunedin, Bielschowsky's NZB BL inbred strain of mice had been found to develop autoimmune haemolytic anaemia [31], spontaneously. To study the genetics, Howie and Helyer crossed NZB mice with the normal NZW strain and found that the F l hybrids spontaneously develop lupus nephritis [32]. Backcross studies showed involvement of three genes in the lupus nephritis, one from the NZB strain and two from the NZW strain [33]. Linkage studies showed none were the expected V genes, one being in the MHC and two being in the neighbourhood of minor histocompatibility antigen genes [34]. Combining the fields of transplantation genetics and autoimmunity, Adams and Knight produced the H Gene Theory [35], which states that histocompatibility antigens, major, minor, and HY, delete nascent lymphocyte clones with complementary antigen receptors and so dictate the immune repertoire, altering the risks of autoimmune diseases. Additionally, germ-line V genes, which provide the initial immune repertoire, influence the risk of autoimmune disease [11].

### 3.3. Addition to the Immune Repertoire by Clonal Deletion

Necessary for the H Gene Theory is explanation of how histocompatibility antigens, by deleting complementary clones, can add to the immune repertoire. It was postulated that microbes bear multiple antigens and that a high-affinity clone for one antigen will preempt reactivity by lower-affinity clones for other antigens on the same microbe. Hence, histocompatibility antigens, by deleting certain clones, can cause others to be added to the immune repertoire and developed by somatic mutation, an *alternative clonal development concept *[11, 25, 36]. 

### 3.4. Confirmation of the H Gene Theory at the Molecular Level

As described below, Ebringer and his colleagues, in the course of discovering the microbial triggers of rheumatoid arthritis and ankylosing spondylitis, have confirmed the H Gene Theory, by finding that these bacterial triggers have two antigens, one resembling the predisposing histocompatibility antigen and one resembling the autoantigen attacked.

### 3.5. The Knight Model of Schizophrenia

Working on Graves' disease, Knight [37] realized that schizophrenia has many similar aetiological features. Accordingly, he postulated that forbidden clones of B lymphocytes develop by somatic mutation and make autoantibodies that act on neuronal cell receptors to affect the function of the brain limbic system. Knight proposed that: the familial aggregation is due to H genes and V genes, and the wait for V gene mutation explains the 50% discordance of monozygous twins and the juvenile grace gap (relative absence in children).

Also explained are:

1. Kety' s adoptive studies [38],
2. Remission and relapse,
3. The rheumatoid arthritis/schizophrenia discordance [39].

Strong confirmation comes from Harrison and Owen's [40] discovery of predisposition by MHC and B-cell light chain V genes, which are immune response genes [11].

### 3.6. A Fourth Category for McKusick's Catalogue

In the light of the Forbidden Clone and H Gene theories, the genetics of autoimmune disease changes from mystery to elegant simplicity. The key is the random element imposed on the genesis of an autoimmune disease by the need for unlucky somatic mutations in lymphocyte V genes.  Table 2 shows that autoimmune disease provides a fourth category to go with dominant, recessive, and sex-linked diseases in McKusick's Catalogue of Mendelian Inheritance in Man [41].

## 4. Microbial Triggers of Autoimmune Diseases

**Rheumatic Carditis and Streptococci**

Before the advent of penicillin, rheumatic fever with lesions of the heart and joints was a frequent autoimmune complication of infections by *β*-haemolytic streptococci of Lancefield Group A. This was because of an antigenic similarity between components of the streptococcus and heart tissue, found by Kaplan and Meyeserian [42]. Today, with such infections therapeutically aborted by penecillin, rheumatic heart disease, once common, has virtually disappeared.

**Glomerulonephritis and Streptococci**

Postinfective glomerulonephritis follows infection by Group A streptococci of multiple M types. This disease also is becoming less frequent due to use of antibiotics.

**Reactive Arthritis**

This has been observed after enteric infection with *Shigella, Salmonella, Yersinia, *and *Campylobacter* and genital infection with *Neisseria gonorrhea. *

**Rheumatoid Arthritis (RA) and *Proteus mirabilis* [43]**

Multiple studies over three decades have found high titres of antibodies against this bacterium in a total of 1,375 RA patients, but not in other diseases or healthy controls, in studies by independent groups in 15 different countries. There was no such elevation in antibodies against 27 other microbial agents. There is evidence that the upper urinary tract is the main source of *Proteus *infection in RA.

**Ankylosing Spondylitis (AS) and *Klebsiella* [44]**

In worldwide studies involving 1330 AS patients and 1191 healthy controls, the AS patients showed significantly increased antibody titres to *Klebsiella.* There is evidence that the gut is the main site of *Klebsiella* infection in AS.

**Schizophrenia and Virus Infection**

Acute schizophrenia has been observed to follow upper respiratory tract virus infections, and G. Knight et al. have assembled much evidence indicating that schizophrenia is an autoimmune disease caused by autoantibodies that react with neuronal receptors influencing the limbic system [45]. Seeking antigenic triggers and their corresponding autoantigen, with excellent technology at the NIH, Laing et al. immunized rabbits with neurotropic strains of influenza virus, inducing autoantibodies to a brain-specific 37 kDa protein [46]. This needs further exploration, as does the whole field of psychotic aetiology, until the presumptive forbidden clones have been demonstrated with the clarity obtained for Graves' disease and myasthenia gravis.

### 4.1. Information from Sequencing Antigens on Triggering Bacteria

Details of the development of the methods used for successful determination of the amino acid sequences of antigens on the autoimmune disease-triggering bacteria, *Proteus mirabilis* and *Klebsiella pneumoniae, *are recorded in the book Rheumatoid Arthritis and Proteus by Ebringer [47]. 

This research provides experimental confirmation, at the molecular level, of the H Gene Theory of the inheritance of the autoimmune diseases, described above, in confirming the speculated presence of multiple antigens on triggering bacteria and alternative clonal development causing the autoimmune disease.

### 4.2. How Histocompatibility Antigens Can Predispose to Autoimmune Diseases

#### 4.2.1. How HLA-DR1/4 Predisposes to Rheumatoid Arthritis [48]

**Figure 1:**
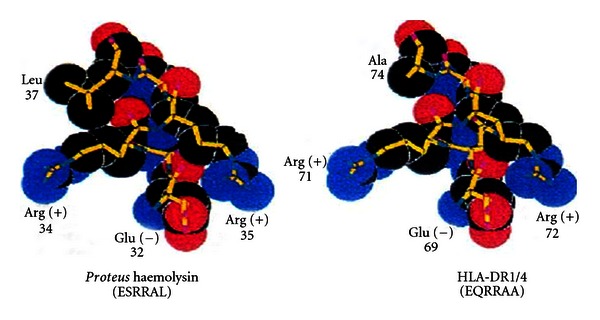
Molecular similarity between histocompatibility antigen HLA-DR1/4 and *Proteus* haemolysin, preventing immune reaction against this bacterial antigen.

Figure 1 shows space-filling models of the amino acid sequences of the histocompatibility antigen, HLA-DR1/4, and the *Proteus mirabilis *haemolysin antigen. Close structural similarity is apparent. This means the immune tolerance imposed by the histocompatibility antigen will extend to this *Proteus *antigen, preventing immune reaction with it.

**Figure 2:**
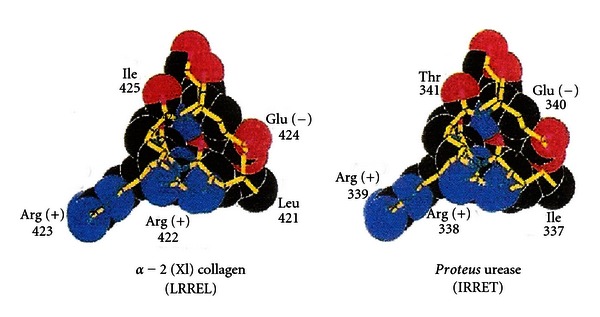
Molecular dissimilarity of *Proteus* urease with HLA-DR1/4, allowing immune reaction with this bacterial antigen, which resembles type X1 collagen, an autoantigen attacked in rheumatoid arthritis.

Figure 2 shows space-filling models of the amino acid sequences of the *Proteus mirabilis* urease antigen and type 11 collagen, an autoantigen attacked in rheumatoid arthritis. The urease antigen is completely different from HLA-DR1/4, so it will not be protected from immune reaction, being free to stimulate development of a forbidden clone reacting with the closely similar type 11 collagen molecule, an autoantigen attacked in rheumatoid arthritis.

#### 4.2.2. How HLA-B27 Predisposes to Ankylosing Spondylitis [49]

**Figure 3:**
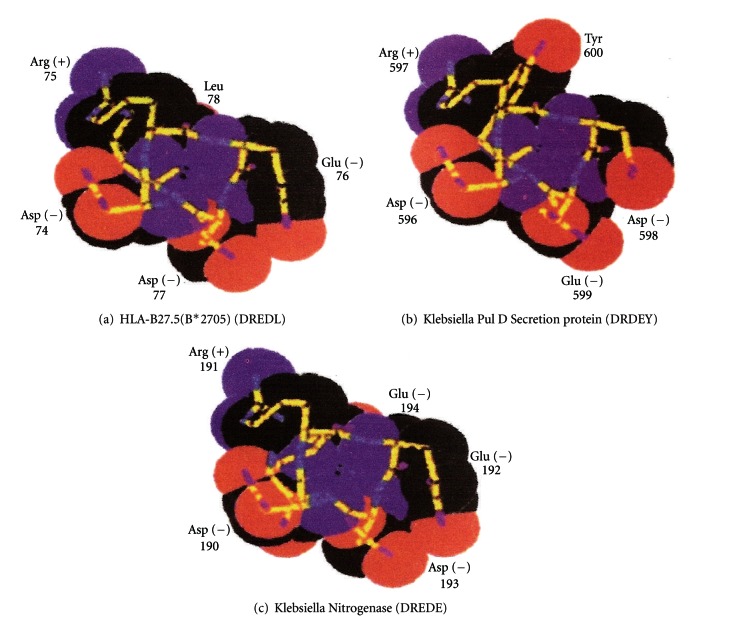
Molecular similarity between HLA-B27 and the nitrogenase reductase peptide of *Klebsiella,* preventing immune reaction, and dissimilarity with the pullanase peptide (Pul D), of *Klebsiella. *This explains how HLA-B27 increases the risk of ankylosing spondylitis.

Figure 3 shows space-filling models of the amino acid sequences of the histocompatibility antigen, HLA-B27, and two antigenic peptides on the bacterium *Klebsiella pneumoniae.* The *Klebsiella* nitrogenase antigen closely resembles HLA-B27, so it will be covered by the tolerance induced by HLA-B27, but the bacterium pullanase peptide is different and able to stimulate development of a forbidden clone, attacking the spine causing ankylosing spondylitisn.

## 5. Four Laws of Autoimmunity

It is now apparent that, in the present state of knowledge, there are 4 laws of autoimmunity, as detailed in Table 3. Recognition of the universality of microbial triggers is a major advance as it shows that all autoimmune diseases are potentially preventable by vaccinations against their microbial triggers.

## 6. Prophylaxis of Autoimmune Diseases

### 6.1. The Poliomyelitis Epidemics

A New Zealand example occurred in 1938 and was reported in the press and observed by Adams, trapped in a boarding school at Masterton. An epidemic of leg paralyses occurred in Christchurch and spread progressively north, from town to town, to Picton, Wellington, Featherstone, then Carterton, the town next to Masterton, engendering great fear. Then, the boy in the bed next to Adams complained of a stiff neck, was taken away, and reported to have polio. Six months later he returned with a paralysed leg. Adams and his 30 school mates were all unaffected.

### 6.2. Was the Paralysis a Rare Autoimmune Complication of Universal Virus Infection?

These events make it probable that the leg paralyses of poliomyelitis were a rare autoimmune complication of virtually universal virus infection, the paralyses probably caused by forbidden clones of cytotoxic T cells which attacked anterior horn neurons, hence the name, “acute anterior poliomyelitis”.

### 6.3. The Lead in Prophylaxis Given by the Polio Vaccines

The Salk (killed) and Sabin (attenuated) polio vaccines have both been brilliantly successful in preventing the polio leg paralyses. This exemplifies how autoimmune diseases in general can be prevented by finding and vaccinating against their microbial triggers.

### 6.4. Finding Microbial Triggers

Ebringer has succeeded in this with rheumatoid arthritis and ankylosing spondalitis, He has pioneered this new field of medical research, developing a whole new technology which needs to be copied in other diseases, especially schizophrenia. Systematic studies of other autoimmune diseases, with collaboration between clinicians and microbiologists, are needed. The American Association of Microbiologists would be an ideal organization for providing this urgently needed knowledge.

## 7. Discussion

Some disease associations are cross-tissue autoimmunity, for example the proptosis of Graves' disease, caused by variants of the thyroid-stimulating autoantibodies that react with receptors on orbital fat cells [9] and diabetic retinopathy [27], probably caused by destruction of retinal pericytes by antigenic variants of the T-cell forbidden clones that destroy the pancreatic islet *β* cells to cause type 1 diabetes.

Many autoimmune diseases, such as Graves' disease, already have satisfactory therapy.

Immunotherapy, by radiological or chemical immune ablation with immune reconstitution by autologous bone marrow cells, pioneered by Farge et al. [50], can be used to save the lives of patients with dangerous autoimmune diseases, such as systemic scleroderma [51].

Selective destruction of forbidden clones could be achieved by isolating their autoantigen (such as the TSH receptor of Graves' disease) and attaching it to a cytotoxic moiety, such as bungarotoxin or ^131^iodine (emitting short-range beta particles), then administering the molecular complex intravenously to destroy the pathogenic clones of plasma cells.

Prevention is better than cure, so finding and countering antigenic triggers of autoimmune diseases is the ideal. Recognition of the universality of microbial triggers of autoimmune diseases is a major advance. It shows how the diseases can be prevented by finding and vaccinating against the triggers. Ebringer has led the way by discovering two major triggers and developing the technology for finding others.

## Figures and Tables

**Table 1:** Classification of immune reactions causing disease [11].

Type I. *A llergy and Anaphylaxis. *Parasite-defence mechanism reacting to non-parasite antigens [52, 53]. Fault: a B lymphocyte IgE clone with specificity for a non-parasite foreign antigen. For example, hay fever, anaphylaxis, gut allergy, skin allergy, contact dermatitis.

Type II. *Serum Sickness and Immune Complex Disease. *Fault: excessive quantity of antigen. This swamps complement-neutralising mechanisms, leading to complement-mediated damage. Anti-microbial immune defence is designed to cope with picogram quantities of antigen, not milligrams of horse serum protein nor micrograms of released intra-cellular protein, such as nuclei [36, 54]. For example, serum sickness following passive immunisation against diphtheria toxin with horse serum, systemic lupus, erythaematosus, lupus nephriti.

Type III. *Autoimmunity. *Fault: forbidden clones, which are anti-microbial lymphocyte clones with accidental host antigen-specificity arising from unlucky somatic mutations in their V genes [19, 36].

Type III B. Diseases caused by B lymphocyte forbidden clones: for example, Graves' disease [9], myasthenia gravis [55], rheumatoid arthritis [56].

Type III T. Diseases caused by T lymphocyte forbidden clones: for example, Diabetes Type 1 [57, 58], experimental autoimmune encephalomyelitis [59], diabetic retinopathy [27], and presumptively Addison's disease, hypoparathyroidism, multiple sclerosis, and other autoimmune diseases with specific parenchymal cell destruction [11].

**Table 2:** A 4th Categorary for McKusick's Catalogue of Mendelian Inheritance in Man [23].

(1) Dominant. *Presence* of the gene causes disease.

(2) Recessive. *Absence* of the gene causes disease.

(3) X-linked. *Sex-linked*, gene on X chromosome.

(4) Autoimmune. *Multiple co-dominant genes* (V genes and H genes) *with incomplete penetrance*, due to need for antigenic triggers and somatic gene mutations in lymphocytes [40, 41].

**Table 3:** Four Laws of Autoimmunity.

The 1st Law of Autoimmunity.All autoimmune diseases are triggered by microbial infections.

The 2nd Law of Autoimmunity.Autoimmunity, the reaction between a host component and the receptor for antigen on a lymphocyte or antibody, occurs due to a semi-random change in the DNA sequence of a lymphocyte V gene.Burnet's Forbidden Clone Theory, BMJ 1959, confirmed by Knight et al JCEM 1986, Adams et al in Thyroid Autoimmunity 1987.

The 3rd Law of Autoimmunity.Histocompatibility antigens, major, minor and HY, protect from autoimmunity with imperfect success, by deleting nascent lymphocyte clones with complementary receptors for antigen. Adams and Knight's H Gene Theory, Lancet 1980.Adams Lancet 1987, Knight and Adams Ciba Foundation Symposium 1982.

The 4th Law of Autoimmune disease. In a population of people, a balanced genetic polymorphism of H and V genes, driven bt reproductive disadvantage, minimises infectious and autoimmune diseases.

A corollary of the 4th Law is that non-pathogenic autoimmunity, having no reproductive disadvantage, occurs much more commonly than pathogenic autoimmunity.

## References

[1] Dubos RJ (1956). *Louis Pasteur*.

[2] Godlee RJ (1917). *Lord Lister*.

[3] Coombs RRA, Gell PGH, RRA Coombs (1962). The classification of allergic reactions underlying disease. *Clinical Aspects of Immunology*.

[4] Himmelweit F (1956). *The Collected Papers of Paul Ehrlich*.

[5] Dameshek W (1965). Autoimmunity: theoretical aspects. *Annals of the New York Academy of Sciences*.

[6] Witebsky E, Rose N, Terplan K, Paine JR, Egan RW (1957). Chronic thyroiditis and autoimmunization. *Journal of the American Medical Association*.

[7] Doniach D, Roitt IM (1957). Auto-immunity in Hashimoto’s disease and its implications. *The Journal of Clinical Endocrinology and Metabolism*.

[8] Adams DD (1958). The presence of an abnormal thyroid-stimulating hormone in the serum of some thyrotoxic patients. *The Journal of Clinical Endocrinology & Metabolism*.

[9] Adams DD (1981). Thyroid-stimulating autoantibodies. *Vitamins and Hormones C*.

[10] Johnson KJ, Chensue SW, Kunkel SL, Ward PA, Rubin E, Faber J (1994). Immunologically mediated tissue injury. *Pathology*.

[11] Adams DD, Knight JG (2003). Principles of autoimmune disease: pathogenesis, genetics and specific immunotherapy. *Journal of Clinical & Laboratory Immunology*.

[12] Jerne NK (1955). The selection theory of antibody formation. *Proceedings of the National Academy of Sciences of the United State*.

[13] Burnet FM (1959). *The Clonal Selection Theory of Acquired Immunity*.

[14] Burnet FM (1959). Autoimmune disease. *British Medical Journal*.

[15] Graves RJ (1838). *Clinical Lectures*.

[16] Harington CR (1933). *The Thyroid Gland*.

[17] Adams DD, Purves HD (1956). Abnormal responses in the assay of thyrotrophin. *Proceedings of the University Otago Medical School*.

[18] Adams DD (1958). The presence of an abnormal thyroidstimulating hormone in the serum of some thyrotoxic patients. *The Journal of Clinical Endocrinology & Metabolism*.

[19] Adams DD, Knight A, Knight JG, Laing P, Pinchera A, Ingbar SH, McKenzie JM, Fenzi GF (1987). Graves' disease; a paradigm for autoimmunity. *Thyroid Autoimmunity*.

[20] Knight J, Laing P, Knight A, Adams D, Ling N (1986). Thyroid-stimulating autoantibodies usually contain only X light chaims: evidence for the forbidden clone theory. *The Journal of Clinical Endocrinology & Metabolism*.

[21] Allison AC, Denman AM, Barnes RD (1971). Cooperating and controlling functions of thymus-derived lymphocytes in relation to autoimmunity. *The Lancet*.

[22] Nachigal D, Zan Bar I, Feldman M (1975). The role of specific suppressor T cells in immune tolerance. *Transplantation Reviews*.

[23] Knight JG, Adams DD (1978). Failure of transferred thymus cells to suppress or prevent autoantibody production in NZB and NZBxNZW mice. *Journal of Clinical and Laboratory Immunology*.

[24] Shevack EM, Paul WE (1999). Organ-specific autoimmunity. *Fundamental Immunology*.

[25] Adams DD, Knight JG, Ebringer A (2010). Autoimmune diseases: solution of the environmental, immunological and genetic components with principles for immunotherapy and transplantation. *Autoimmunity Reviews*.

[26] Klintworth GK, Rubin E, Farber JL (1988). The eye. *Pathology*.

[27] Adams DD (2008). Autoimmune destruction of pericytes as the cause of diabetic retinopathy. *Journal of Clinical Ophthalmology*.

[28] Adams DD (2011). Why the histocompatibility system exists and how transplant surgeons can xenograft without rejection. *An International Journal of Medicine*.

[29] Adams DD (1987). Protection from autoimune disease as the third function of the major histocompatibility gene complex. *The Lancet*.

[30] Zinkernagel RM, Doherty PC (1974). Restriction of in vitro T cell mediated cytotoxicity in lymphocytic choriomeningitis within a syngeneic or semiallogeneic system. *Nature*.

[31] Bielschowsky M, Helyer BJ, Howie JB (1959). Spontaneous anaemia in mice of the NZB/BL strain. *Proceedings of the University Otago Medical School*.

[32] Howie JB, Helyer BJ (1968). The immunology and pathology of NZB mice 1. *Advances in Immunology C*.

[33] Knight JG, Adams DD (1978). Three genes for lupus nephritis in NZB X NZW mice. *Journal of Experimental Medicine*.

[34] Knight JG, Adams DD (1981). Genes determining autoimmune disease in New Zealand mice. *Journal of Clinical and Laboratory Immunology*.

[35] Knight JG, Adams DD (1982). The genetic basis of autoimmune disease. *Ciba Foundation Symposium*.

[36] Adams DD, Davies TF (1983). Autoimmune mechanisms. *Autoimmune Endocrine Disease*.

[37] Knight JG (1982). Dopamine-receptor-stimulating autoantibodies: a possible cause of schizophrenia. *The Lancet*.

[38] Kety SS (1983). Mental illness in the biological and adoptive relatives of schizophrenic adoptees: findings relevant to genetic and environmental factors in etiology. *American Journal of Psychiatry*.

[39] Mellsop GW, Koadlow L, Syme J, Whittingham S (1974). Absence of rheumatoid arthritis in schizophrenia. *Australian and New Zealand Journal of Medicine*.

[40] Harrison PJ, Owen MJ (2003). Genes for schizophrenia? Recent findings and their pathophysiological implications. *The Lancet*.

[41] McKusick VA (1978). *Mendelian Inheritance in Man*.

[42] Kaplan Mh, Meyeserian M (1962). An immunological cross-reaction between group A streptococcal cells and human heart tissue. *The Lancet*.

[43] Rashid T, Ebringer A (2007). Rheumatoid arthritis is linked to Proteus—the evidence. *Clinical Rheumatology*.

[44] Ebringer A, Rashid T, Wilson T, Ptaszynska T, Fielder M (2006). Ankylosing spondylitis, HLA-b27, and Klebsiella—an overview: proposal for early diagnosis and treatment. *Current Rheumatology Reviews*.

[45] Knight JG, Knight A, Pert CB, Helmchen H, Henn FA (1987). Is schizophrenia a virally-triggered anti-receptor autoimmune disease?. *Biological Perspectives of Schizophrenia*.

[46] Laing P, Knight JG, Hill JM (1989). Influenza viruses induce autoantibodies to a brain-specific 37-kDa protein in rabbit. *Proceedings of the National Academy of Sciences of the United States of America*.

[47] Ebringer A (2011). *Rheumatoid arthritis and Proteus*.

[48] Wilson C, Ebringer A, Ahmadi K (1995). Shared amino acid sequences between major histocompatibility complex class II glycoproteins, type XI collagen and Proteus mirabilis in rheumatoid arthritis. *Annals of the Rheumatic Diseases*.

[49] Ebringer A, Rashid T, Wilson C, Ptazynska T, Fielder M (2006). Ankylosing spondylitis, HLA-B27 and Klebsiella pneumoniae—an overview proposed for early diagnosis and treatment. *Current Rheumatology Reviews*.

[50] Farge D, Passweg J, Van Laar JM (2004). Autologous stem cell transplantation in the treatment of systemic sclerosis: report from the EBMT/EULAR registry. *Annals of the Rheumatic Diseases*.

[51] Englert H, Katelaris C, McGill N, Schrieber L, Moore J (2005). “Grape-sultana” sign represents a favourable response to aggressive treatment of early diffuse systemic scleroderma. *Internal Medicine Journal*.

[52] Jarrett EE, Miller HR (1982). Production and activities of IgE in helminth infection. *Progress in Allergy*.

[53] Bell RG (1996). IgE, allergies and helminth parasites: a new perspective on an old conundrum. *Immunology and Cell Biology*.

[54] Adams DD, Dawkins RL, Christiansen FT, Zilko PJ (1982). Systemic lupus erythaematosus: a simple concept of the pathogenesis and its genetic basis. *Immunogenetics in Rheumatology*.

[55] Lindstrom J, Shelton D, Fujii Y (1988). Myasthenia gravis. *Advances in Immunology*.

[56] Ebringer A, Rashid T, Wilson C (2010). Rheumatoid arthritis, *Proteus*, anti-CCP antibodies and Karl Popper. *Autoimmunity Reviews*.

[57] Sherwin RS, Goldman L, Bennett JC (2000). Diabetes mellitus. *Cecil Textbook of Medicine*.

[58] Sutherland DE, Goetz FC, Sibley RK (1989). Recurrence of disease in pancreas transplants. *Diabetes*.

[59] Ben-Nun A, Wekerle H, Cohen I (1981). The rapid isolation of clonable antigen-specific T lymphocyte lines capable of mediating autoimmune encephalomyelitis. *European Journal of Immunology*.

